# Simple Strategy for Scalable Preparation Carbon Dots: RTP, Time‐Dependent Fluorescence, and NIR Behaviors

**DOI:** 10.1002/advs.202104278

**Published:** 2021-12-28

**Authors:** Jianliang Bai, Guojun Yuan, Xu Chen, Lu Zhang, Yaqing Zhu, Xinyu Wang, Lili Ren

**Affiliations:** ^1^ School of Chemistry and Chemical Engineering Southeast University Nanjing 211189 China

**Keywords:** carbon dots, dispersion‐induced redshift, large‐scale preparation, near‐infrared emission, room‐temperature phosphorescence

## Abstract

Transforming carbon dots (CDs) fluorescent materials into smart materials with complex functions is a topic of great interest to nanoscience. However, designing CDs with regulating fluorescence/phosphorescence that can be visually monitored with the environment changes in real‐time remains a challenge. Here, a very simple strategy, one‐step solvent‐free catalytic assistant strategy, which is low cost, facile, environment‐friendly, and high throughput, is put forward. Hydrogen bond is used to manipulate nanostructure of CDs, and the obtained carbon dots (M‐CDs) show a series of attractive properties including matrix‐free room‐temperature phosphorescence, time‐dependent fluorescence, and near‐infrared emissive characteristics. Different from the traditional aggregation caused quenching or aggregation‐induced emission fluorescent materials, M‐CDs exhibit unprecedented and unique dispersion induced redshift fluorescence phenomenon, promoting the studies of fluorescence from static to dynamic. The causes of this phenomenon are further analyzed in detail. As a kind of intelligent fluorescent materials, this new designed CDs greatly enrich the basic recognition of CDs by illustrating the relationship between redshift fluorescence behaviors and the dispersion states, and may provide with an opportunity for solid‐state fluorescent materials, anti‐counterfeiting, cellular imaging, and hopefully many others.

## Graphical abstract

Illustration of simple solvent‐free catalytic assistant strategy for scalable preparation smart and versatile carbon dots (matrix‐free room‐temperature phosphorescence, time‐dependent fluorescence, and near‐infrared behaviors).

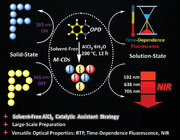

## 1 Introduction

Smart optical materials are materials that can respond dynamically and selectively to the changes of the external environment they are exposed to.^[^ ^1^ ^]^ Over the past three decades, much research has been devoted to developing intelligent optical materials that can sense‐and‐respond to external chemical and physical perturbations. Prospectively, smart optical materials with dynamically properties could be significantly superior for applications.^[^ ^2^ ^]^

Recently, carbon dots (CDs) as a novel kind of luminescent nanomaterials have attracted great interest of researchers due to their unique advantages such as environmentally friendly preparation,^[^ ^3^ ^]^ high photostability,^[^ ^4^, ^5^ ^]^ numerous precursor sources,^[^ ^6^, ^7^ ^]^ and low toxicity.^[^ ^8^ ^]^ So far, CDs have been applied in various fields including drug delivery, bioimaging, sensor, and optoelectronic devices.^[^ ^9^, ^10^, ^11^, ^12^ ^]^ To further expand their applications, it is meaningful to produce smart CDs that can display time‐dependent fluorescence emission,^[^ ^13^, ^14^ ^]^ but this is more technically challenging and has not been widely reported yet. The main reason is that most CDs undergo aggregation‐caused quenching (ACQ) in the solid state, which is ascribed to the direct *π*–*π* interactions and fluorescence resonance energy transfer (FRET), limiting their application in a broader of fields.^[^ ^15^, ^16^, ^17^, ^18^, ^19^, ^20^, ^21^, ^22^ ^]^ Up to now, a common way to obtain the solid‐state FL emission of CDs is to disperse CDs into solid matrices (like polymers,^[^ ^19^, ^20^, ^21^, ^22^, ^23^ ^]^ starch,^[^ ^17^, ^24^ ^]^ inorganic salts,^[^ ^25^, ^26^, ^27^ ^]^ mesoporous materials,^[^ ^28^, ^29^ ^]^ cellulose nanofiber,^[^ ^30^ ^]^ and agar^[^ ^31^ ^]^) to secure sufficient distance between CDs.^[^ ^32^, ^33^, ^34^ ^]^ However, if too many CDs are introduced into the solid matrices, the ACQ effect still exists, and only 0.1–1 wt% of CDs can be used in the supporting media.^[^ ^35^, ^36^, ^37^ ^]^ Therefore, it is necessary to develop the matrix‐free solid‐state luminescent CDs.

Unlike the ACQ property of nanomaterials, aggregation‐induced emission (AIE) has attracted more and more attention in recent 20 years.^[^ ^38^, ^39^, ^40^ ^]^ Compared to the conventional fluorophores, materials with AIE properties are known to show intense fluorescence only in aggregated or even solid state.^[^ ^41^, ^42^ ^]^ Yang and co‐workers also discovered a series of carbonized polymer dots (CPDs),^[^ ^43^, ^44^ ^]^ as a new kind of CDs, inherit the merits of CDs‐based composite materials, possessing both the high fluorescent properties of CDs and the matrix effects permeated by polymers, which can replace covalent bonds for supramolecular interactions and then greatly strengthen the fixation.^[^ ^43^, ^44^, ^45^ ^]^ Both AIE materials and CPDs were obtained in solutions reaction. And the related researches are mainly focused on the optical properties of fluorophores from nonluminescent in the dispersed state to emissive in the aggregated state or study their optical properties in the solid state directly. In contrast to AIE materials and CPDs, is it possible to develop a solvent‐free method to obtain luminous CDs in aggregate state directly? And then the optical properties of CDs from solid state to dissolved state can be further studied. Of course, the noncovalent interactions among carbon nanoparticles are the key factor for tuning fluorescence.

**Scheme 1:**
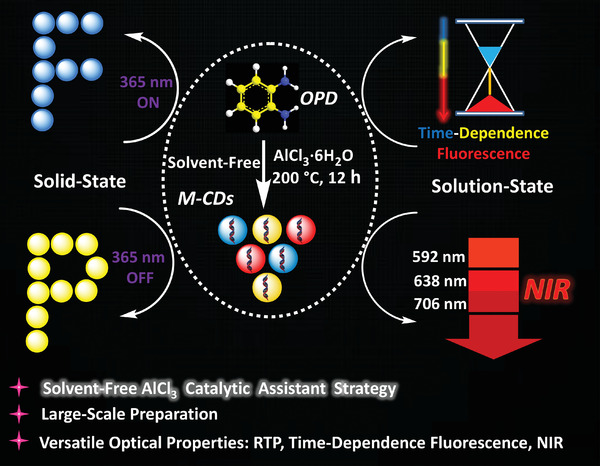
Schematic illustration of the solvent‐free catalytic assistant strategy to prepare M‐CDs and its versatile optical properties.

Here, we designed a dynamically evolved and full‐color‐tunable chameleon‐like CDs from solid to dissolved states, which were named magic carbon dots (M‐CDs). Just as shown in **Scheme** **1**, unlike previous luminescent CDs, M‐CDs are obtained via a simple solvent‐free catalytic assistant strategy, with matrix‐free room‐temperature phosphorescence (RTP), time‐dependent fluorescence and near‐infrared (NIR) emissive characteristics. More importantly, the reaction volume can be flexibly scaled up or down according to the demand; therefore, the M‐CDs have great potential for large‐scale synthesis. These characteristics may provide potential applications for CDs in anti‐counterfeiting, cellular imaging, and smart devices, among others.

## 2 Results and Discussion

**Figure 1:**
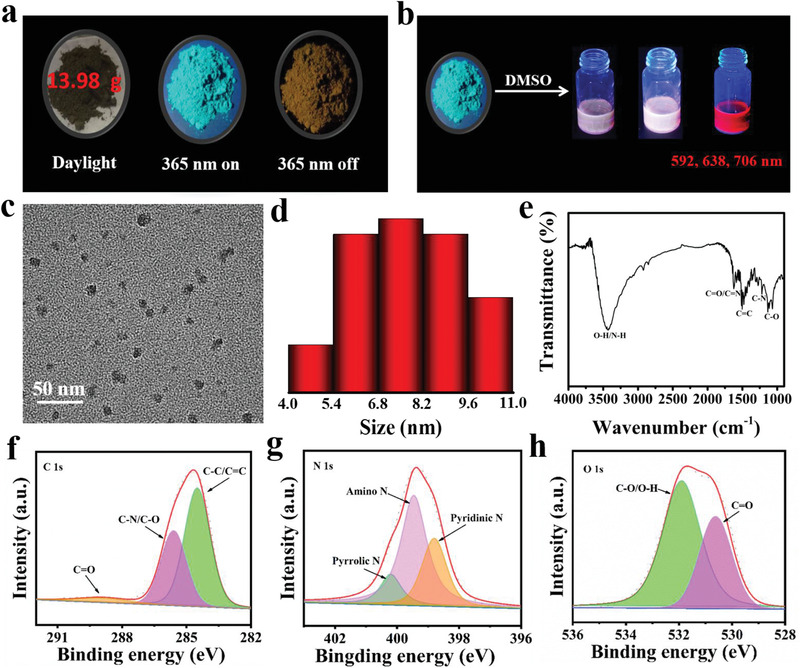
a,b) The photographs show versatile optical properties of M‐CDs (matrix‐free RTP, time‐dependent fluorescence, and NIR). c) TEM image of M‐CDs. d) Corresponding size distribution of M‐CDs. e) FTIR spectrum of M‐CDs. f–h) High‐resolution XPS C 1s, N 1s, and O 1s spectra of M‐CDs.

As shown in Scheme 1 and Figures S1 and S2 (Supporting Information), the target M‐CDs were easily synthesized through a simple in situ solvent‐free catalytic assistant strategy via directly heating of *o*‐phenylenediamine (OPD) and AlCl_3_·6H_2_O. As the reaction temperature rises, AlCl_3_·6H_2_O first decomposed into AlCl_3_ (g) and H_2_O (g), which could provide more catalytic sites to help form conjugated region, just like heterogeneous catalytic process. Under the catalytic action of AlCl_3_, a polymerization took place between OPD at high temperature and high pressure. At the same time, the sublimation of the catalyst facilitates the construction of more hydrogen bonds among the carbon nanoparticles, similar to increase the effective collisions that occur in the chemical reaction process. Moreover, the trace amount of water will cause the surface of the M‐CDs to form a part of the —OH and C═O groups under high temperature conditions. As expected, the obtained M‐CDs present a series of fascinating phenomena. In the solid state, it shows blue emission under ultraviolet light (365 nm) and yellow RTP after ceasing the light source; in the solution state, it shows visually time‐dependent fluorescence color changes (from blue to red) and NIR emission (**Figure** **1**a,b). Moreover, in view of the facile manual grinding and one‐step solvent‐free catalytic assistant strategy, gram‐scale preparation of products can be readily achieved in laboratory (Figure S3, Supporting Information), which means that this method has great potential of mass production in industrial applications.

To further determine the nature of M‐CDs, their morphologies were characterized by transmission electron microscopy (TEM). As shown in Figure 1c,d, the TEM image reveals that M‐CDs exhibit well dispersed nearly spherical shape and the average diameter of CDs is 7.74 nm. To explore the functional group composition of M‐CDs, ^1^H NMR spectrum was shown in Figure S4 (Supporting Information). The intense signals centered at 7.99 ppm are corresponding to the aromatic H. Other H signals —OH (11.70 ppm), —NH (8.25–8.27 ppm), and —NH_2_ (6.34–6.49 ppm) are also recorded. The FTIR spectrum of the M‐CDs was shown in Figure 1e. The absorption peak near 3432 cm^–1^ can be assigned to the stretching vibrations N—H and O—H.^[^ ^46^ ^]^ The peaks located at 1625 and 1580 cm^–1^ correspond to the stretching vibration of C═O and C═N, respectively.^[^ ^35^, ^47^ ^]^ Three bands at the wavenumbers of 1503, 1206, and 1121 cm^–1^ are attributed to C═C, C—N, and C—O stretching frequencies, respectively.^[^ ^48^ ^]^ To further verify the above results of FTIR analyses, X‐ray photoelectron spectra (XPS) of the M‐CDs were measured (Figure 1f–h and Figure S5, Supporting Information). The high‐resolution C 1s XPS spectrum is shown in Figure 1f, and the band can be deconvoluted three peaks at 284.5 eV (C—C/C═C), 285.6 eV (C—O/C—N), and 288.9 eV (C═O/C═N).^[^ ^41^, ^48^, ^49^, ^50^ ^]^ The N 1s spectrum reveals three distinct peaks at 398.8 eV (pyridinic Ns), 399.6 eV (amino Ns), and 400.2 eV (pyrrolic Ns) (Figure 1g).^[^ ^41^, ^48^, ^49^, ^50^ ^] ^The O 1s spectrum shows two peaks at 530.6 and 531.9 eV, which are ascribed to C═O and C—O/O—H functional groups, respectively (Figure 1h).^[^ ^41^, ^48^, ^49^, ^50^ ^]^ The above results reveal that the surface of M‐CDs is rich in amino and hydroxyl functional groups, which can effectively produce hydrogen bonds among carbon nanoparticles, just like melamine and cyanuric acid.^[^ ^51^, ^52^ ^]^ This can also be demonstrated in the results of comparative experiment and FTIR comparative characterization (see Figure S6, Supporting Information, for details).^[53,54]^ In addition, as shown in Figure S2 (Supporting Information), the obvious polymer structure was formed during the synthesis of M‐CDs.

**Figure 2:**
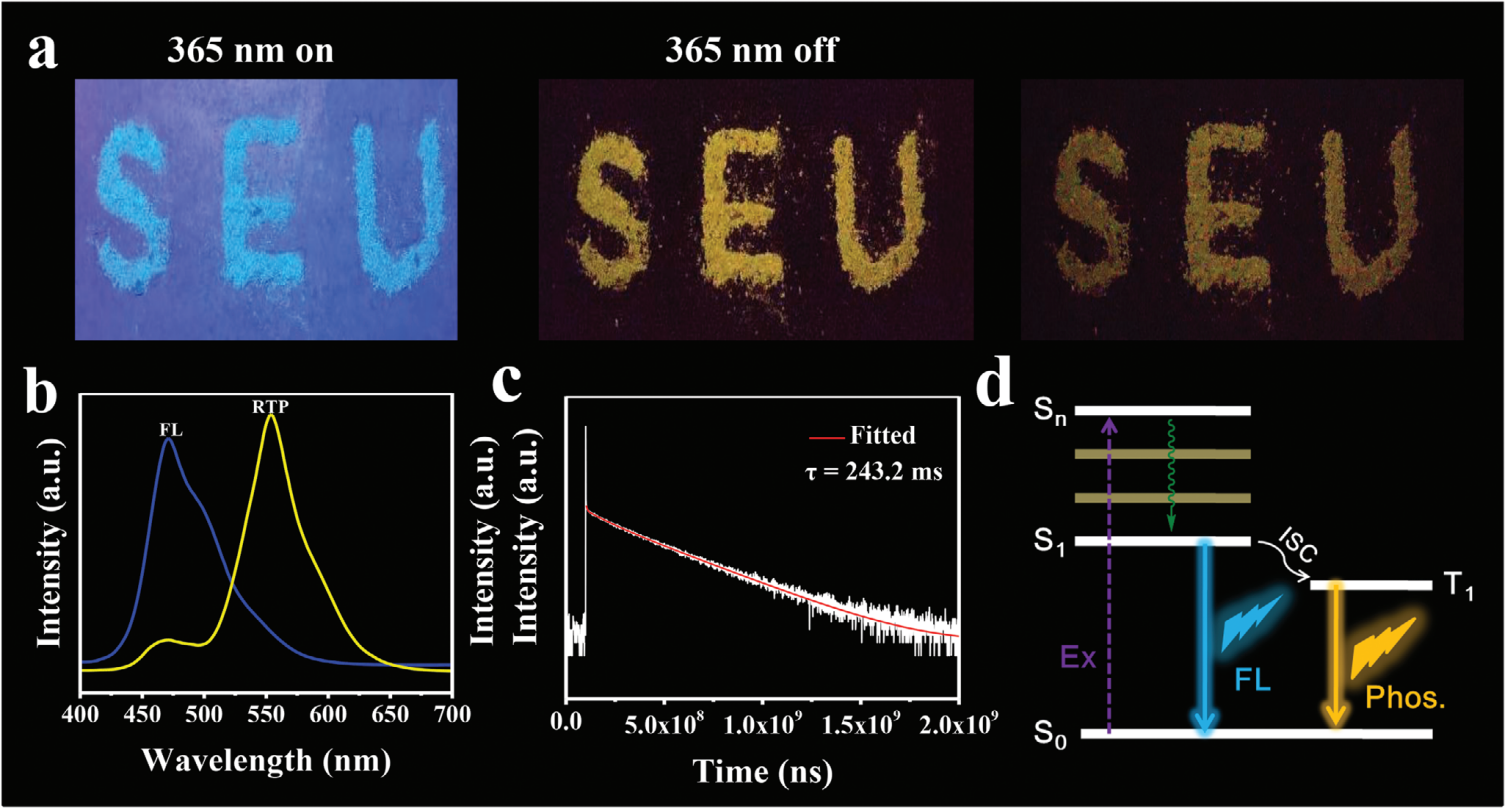
a) Photographs of the M‐CDs powder under UV lamp (365 nm) on and off, respectively. b) Fluorescence (blue line) and phosphorescence (yellow line) spectra of M‐CDs, excited at 365 and 364 nm, respectively. c) RTP decay spectrum of the M‐CDs powder, recorded at emission wavelengths of 554 nm under excitation at 276 nm. d) Illustration of fluorescence (FL) and phosphorescence (phos.) processes of M‐CDs.

For the solid‐state optical properties of M‐CDs, we focused on the research of fluorescence and phosphorescence of the samples. The obtained M‐CDs appear as charcoal black powders (Figure 1a), emitting blue light under ultraviolet light (365 nm) (**Figure** **2**a). Yellow phosphorescence is observed after ceasing the light source, which lasts about 1 s (Figure 2a and Movie S1, Supporting Information). Systematic optical and structural characterizations reveal that the purposefully designed N‐element doped covalent crosslinked polymer frame and hydrogen bond network structure are the keys to observe blue fluorescence and yellow RTP.^[^ ^51^, ^52^ ^]^ The FL and RTP emission of M‐CDs arise from the certain sub‐fluorophores containing N element and immobilization of the excited triplet species in M‐CDs, respectively (Figure 2d). In addition, trace amount of Al^3+^ can promote electron transfer and is also an important factor for RTP.^[^ ^55^ ^]^ The fluorescent quantum yield (QY) and phosphorescent QY of the M‐CDs powder are 3.2% and 2.8%, respectively.

To further understand the RTP characteristics of the M‐CDs, phosphorescence and afterglow decay spectra were also measured (Figure S7, Supporting Information, and Figure 2c). The phosphorescence spectrum appears as obvious peaks located at ≈553 nm (Figure S7, Supporting Information). The corresponding phosphorescence spectra of the M‐CDs powder show that their emission maxima located at ≈553 nm does not shift with the excitation wavelength changing from 276 to 364 nm, which imply M‐CDs without the excitation wavelength‐dependence of RTP (Figure 2b and Figure S7, Supporting Information). As shown in Figure 2c, the spectrum has been fitted with a tri‐exponential function with lifetime components of 5.2, 95.8, and 251.9 ms, respectively (Table S1, Supporting Information). These results hint that the prepared M‐CDs occupy multiple decay channels. According to the following equation^[^ ^51^ ^]^

(1) $$\tau_{\mathrm{avg}}=\Sigma \alpha i\tau i^{2}/\Sigma \alpha i\tau i$$

An average decay time was calculated to be 243.2 ms under excitation at 276 nm. We speculate that the M‐CDs with RTP may be attributed to the formation of polymers‐like network structure and the multiple hydrogen bonds between them, which may play an important role in the acquisition of afterglow. The hydrogen‐bond framework structure plays an irreplaceable role in stabilizing the excited triplet state, decreasing the nonradiation transitions of triplet excitons. Besides, N‐doped content promotes the n‐*π* transition and hence facilitates the spin‐forbidden transfer of singlet‐to‐triplet excited states through intersystem crossing to populate triplet excitons. Therefore, abundant hydrogen bond interactions and N‐doped content is good for long‐lived excited states and ultralong afterglow.^[^ ^51^ ^]^

**Figure 3:**
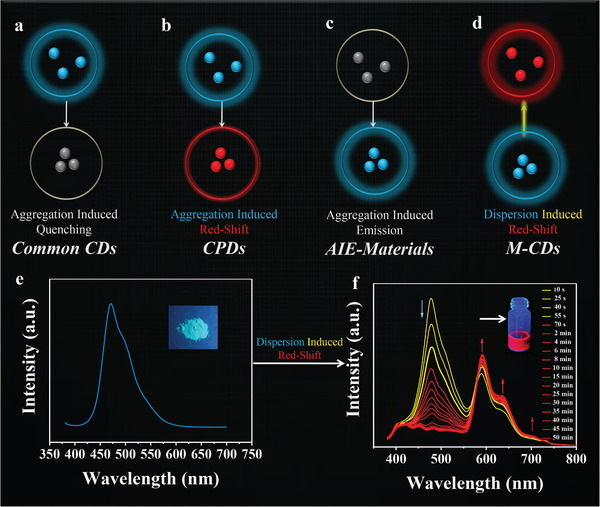
Fluorescence principle of a) common CDs, b) CPDs, c) AIE‐materials, and d) M‐CDs. e,f) Fluorescence emission spectra recorded immediately after dispersed in DMSO solution.

**Figure** 3a‐d shows the FL changes of different kinds of fluorescent materials between the dispersed and aggregated states. As we mentioned before, most CDs face the problem of ACQ effects in the solid state (Figure 3a).^[^ ^15^, ^16^, ^17^, ^18^, ^19^, ^20^, ^21^, ^22^ ^]^ The CPDs resolve this problem at some level, but often encounter redshifted emission with noticeable color change compared to solution‐state fluorescence (Figure 3b).^[^ ^37^, ^43^, ^44^, ^45^ ^]^ In previous reports, nonconjugated small molecules and linear polymers were chosen to prepare CPDs, which could avoid any possible conjugated components.^[^ ^43^, ^44^, ^45^ ^]^ On the contrary, as shown in Figure 3e, M‐CDs give an emission maximum at about 472 nm (blue solid‐state fluorescence) under the excitation of 365 nm UV light, which prepared via “bottom‐up” reaction using conjugated small molecules as precursor, may appear redshifted emission in the solution state in theory. As expected, the hydrophobic M‐CDs dissolve very easily in DMSO (The same phenomenon happens in DMF solution), and the solution displays a time‐dependent fluorescence. The color visually transforms from blue to yellow to red, which shows chameleonic adaptive expression to some extent. When the M‐CDs were dissolved in DMSO, both the FL wavelength and the intensity were progressively varied, which corresponding response time (color visually changing from the initial blue to the final red) was ≈15 min, as revealed by the optical images shown in Figure 3e,f and Movie S2 (Supporting Information), suggesting a time response in solutions. Both RTP and time‐dependent fluorescence features also bring additional encryption possibilities for future information processing.

In addition, as an electron‐accepting group with the electronegativity, C═O has an electron‐withdrawing tendency, which can decrease the energy level between highest occupied molecular orbital (HOMO) and lowest unoccupied molecular orbital (LUMO).^[^ ^55^ ^]^ As shown in Figure 3f, upon excitation with 365 nm radiation, there are three obvious fluorescence emission peaks in the red region at 592, 638, and 706 nm with a tail extending to 800 nm, which cover the whole red region and even enter into NIR‐I region. The absolute fluorescent QY of the M‐CDs in DMSO is 57%. So, M‐CDs have significant potential for practical application in living systems.^[^ ^48^, ^56^, ^57^ ^]^

**Figure 4:**
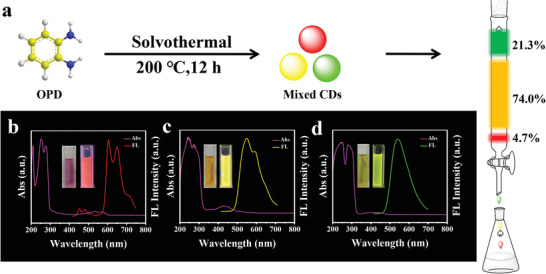
a) One‐pot synthesis and purification route for CDs‐1‐3 (The as‐prepared mixture was separated on a silica column to obtain fractions of CDs exhibiting red, yellow, and green fluorescence). b–d) UV–vis absorption and fluorescence spectra for CDs‐1‐3. Insets in (b–d): the images of the CDs‐1‐3 in ethanol solvents under daylight and UV lamp (365 nm), respectively.

**Figure 5:**
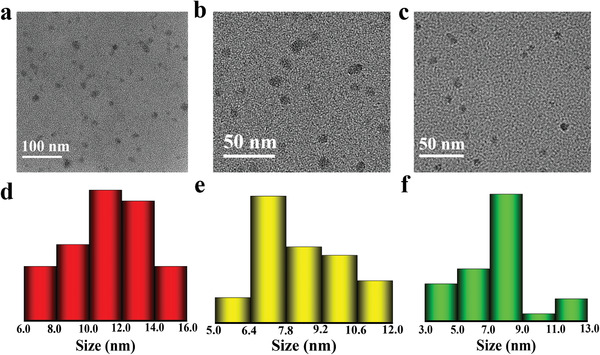
a–c) TEM images and d–f) corresponding size distributions of CDs‐1‐3.

Solid‐state FL, RTP, and time‐dependent redshift were observed in emission, which is notably rare for single‐component luminescent materials. To explore the causes of these phenomena for M‐CDs, several experiments were carried out. As shown in Figure S8 (Supporting Information), phenylenediamine isomers as precursors are considered to be good choices for CDs preparation,^[^ ^3^ ^]^ and *p*‐phenylenediamine (PPD) and OPD are usually used for the preparation of long‐wavelength emissive CDs.^[^ ^3^, ^48^, ^58^, ^59^, ^60^ ^]^ In general, purification by column chromatography method usually results in CDs of varying colors and different proportions.^[^ ^59^ ^]^ Hence, this time, using OPD as the precursor and ethanol as the solvent, red (CDs‐1), yellow (CDs‐2), and green (CDs‐3) color FL CDs were synthesized by one‐pot solvent thermal synthesis, and purified by column chromatography (**Figures** **4** and **5**). The deep‐red luminescent CDs (CDs‐1) has two emission centers (Figure 4b), which is consistent with the performance of CDs‐4 prepared by PPD (Figure S9, Supporting Information).^[^ ^61^ ^]^

More surprisingly, the FL intensity of the two emission centers varied with the dispersion and aggregation of the CDs‐1. It should be noted that the common feature of the CDs of “phenylenediamine series” is that it is easy to precipitate when placed for a long time.^[^ ^60^ ^]^ As shown in Figure S10 (Supporting Information), after 15 d, a small amount of precipitation apperard in a the clear CDs‐1 ethanol solution. At this time, CDs‐1 ethanol solution exhibited blue fluorescence under ultraviolet light after shaking. The FTIR data in Figure S11a (Supporting Information) illustrate the presence of O—H/N—H (3438 cm^–1^), C═O/C═N (1633 cm^–1^), C═C (1508 cm^–1^), and C—N (1213 cm^–1^) stretching vibrations of the red emissive component (surface state). The characteristic D and G bands of Raman spectrum are located at 1390 and 1524 cm^–1^, respectively, which proves the existence of core‐state (Figure S11b, Supporting Information).^[^ ^62^ ^]^ Figure S12 (Supporting Information) exhibits the FL mechanism of the CDs‐1 in dispersed state and aggregated state. When the CDs are in dispersed state, they display red emission from surface states, which consists of *π*‐conjugated polymer fluorescent molecules. However, in aggregated state, *π*‐conjugated domain emission center was suppressed, leading to blue‐emission‐dominant FL feature from core state.^[^ ^63^ ^]^

**Figure 6:**
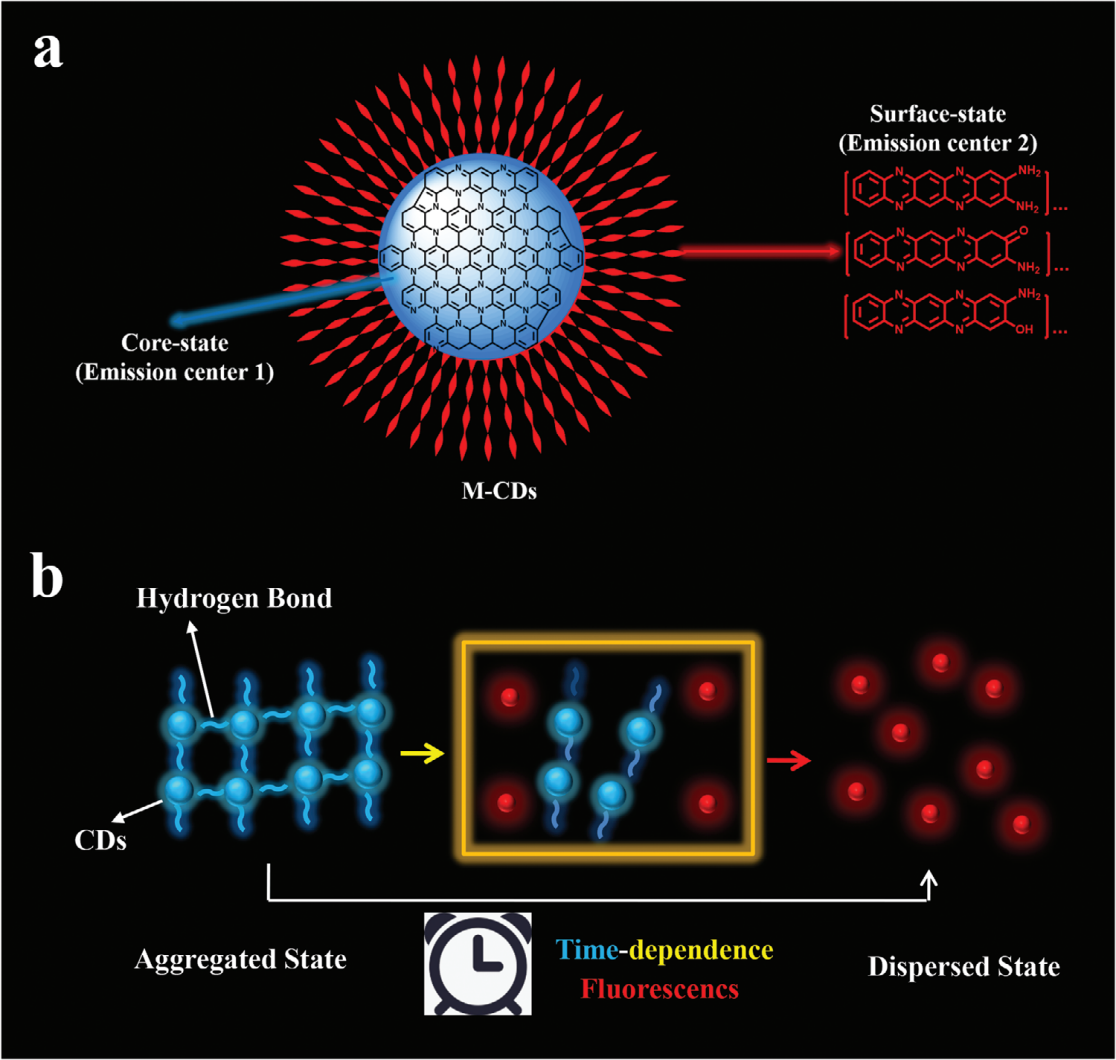
a) The rational structure of M‐CDs. b) Schematic of possible emission mechanism of dispersion induced redshift fluorescence with time dependent.

From the above results, the versatile optical properties of M‐CDs can be rationally explained in terms of the proposed model in **Figure** **6**a,b. The M‐CDs powders possess fluorescence and RTP due to more hydrogen bonding interactions just like some AIE materials.^[^ ^64^ ^]^ The surface‐state emission center of M‐CDs suffer from self quenching in the aggregated state due to direct *π*–*π* interactions; however, the core‐state emission center can emit blue fluorescence. However, unlike common pure organic materials with AIE (Figure 3c) characteristic, CDs with AIE characteristic can adjust color between aggregation and dispersion attributed to their unique core states and surface states. The M‐CDs aggregates generate blue emission and the monomers contribute to the red emission, which reveals the relationship between the M‐CDs’ fluorescence mechanism and their dispersed state. After dispersed in the solvent, as time goes, numerous hydrogen bonding interactions among CDs are destroyed, the hydrogen bonding interactions between CDs and solvent increases, and then the direct *π*–*π* interactions from surface states decrease, leading to the obvious time‐dependent redshift in emissions (Figure 6b). To verify the above analyses, UV–vis spectra were measured. As shown in Figure S13a (Supporting Information), M‐CDs aggregates show very broad absorption bands across purple light to NIR regions (400–800 nm), suggesting strong *π*–*π* interactions from surface states in aggregates, leading to significant aggregation induced red fluorescence quenching. In contrast, as shown in Figure S13b (Supporting Information), M‐CDs in DMSO solution exhibit a much narrower absorption band, indicating the dispersion induced red fluorescence from the surface states.^[^ ^65^, ^66^ ^]^ The direct and dynamic evidence strongly illustrates the change of fluorescence from the aggregated state to the dispersed state (Figures 3e,f and 6b). Thus, this work will provide a strategy to prepare unprecedented dispersion induced redshift fluorescence materials, open a new view to develop CDs with AIE characteristics for fluorescent color regulation.

In addition, the metal chloride (such as FeCl_3_, AlCl_3_, and ZnCl_2_) turned out to be a potential catalyst for the rapidly and selectively preparation of red fluorescent CDs.^[^ ^48^, ^67^, ^68^, ^69^ ^]^ As shown in Figure S14 (Supporting Information), obviously, the addition of AlCl_3_ effectively promoted the increase of polymerization process and *π*‐conjugated region, making it easier to form red emission CDs. To further confirm the effect of the catalyst on the optical properties of CDs, OPD and CuCl_2_·2H_2_O were selected as the comparative experiment, and the prepared CDs were named M′‐CDs. To ensure the effective comparison, we used the same preparation conditions, just like the synthesis of M‐CDs (see the Supporting Information). It is apparent that M′‐CDs exhibit weak FL and RTP compared with M‐CDs in the solid state (Figure S15a, Supporting Information). During the reaction, the solid CuCl_2_ (200 °C) may provide fewer effective collisions among nanoparticles and decrease the number of hydrogen bonds, thereby weakening the RTP performance. In the solid state, M′‐CDs display similar fluorescence behavior as M‐CDs, the FL spectrum appears as an emission maximum at about 471 nm under the excitation of 365 nm UV light (Figure S16a,b, Supporting Information). In the DMSO solution, as shown in Figure S15b (Supporting Information), the UV–vis absorption spectrum of M′‐CDs demonstrate that they have extraordinarily broad absorption from the UV–vis to the NIR region (400–800 nm) and exhibit main absorption peaks at 521, 555, and 630 nm with a tail extending to 800 nm. The strong absorptions at 555 and 630 nm can be assigned for C═N absorption of the pyridinic‐N.^[^ ^56^ ^]^ These results imply that we may be able to choose catalyst according to optical requirements.

## 3 Conclusions

In a word, this simple and scalable solvent‐free catalytic assistant strategy builds a bridge between nanometer luminescent materials and catalytic science. The chameleon‐like M‐CDs show versatile optical properties of fluorescence and phosphorescence, which make it possible for M‐CDs to be used as smart materials. Through changing the luminous environment, phosphorescence or fluorescence of M‐CDs can adapt to environment changes in real‐time. In the solid state, the polymer‐liked structures with amino, alongside the enhanced emission effect of hydrogen bonds crosslink were confirmed to both contribute to the generation of fluorescence and RTP of M‐CDs. In the solution state, hydrogen bonding interaction can act as a tool to manipulate nanostructure of CDs, the unexpected full‐color evolution resulting from adaptive expression of M‐CDs as time goes on. As a seminal work, this study may stimulate scientific community to further exploit the versatile applications in solid‐state fluorescent materials, cellular imaging and anti‐counterfeiting. Additionally, these findings show opportunities for the development of smart CDs with dynamically controllable responsive behavior in advanced optical applications.

## 4 Experimental Section

For this part, please see more details in the Supporting Information.

## Conflict of Interest

The authors declare no conflict of interest.

## Supporting information

Supporting Information

Supplemental Movie 1

Supplemental Movie 1

## Data Availability

Research data are not shared.
