# Supplementary material for: Simple Strategy for Scalable Preparation Carbon Dots: RTP, Time‐Dependent Fluorescence, and NIR Behaviors
Source: Adv Sci (Weinh). 2021 Dec 28;9(5):2104278. doi: 10.1002/advs.202104278 (PMC8844479; doi:10.1002/advs.202104278)
Supplement: Supplementary file 1 — Supporting Information [file ADVS-9-2104278-s002.pdf]

© 2020 Wiley-VCH GmbH

ADVANCED SCIENCE Open Access

Supporting Information

for *Adv. Sci.,* DOI: 10.1002/advs.202104278

Simple Strategy for Scalable Preparation Carbon Dots: RTP, Time-dependence Fluorescence and NIR Behaviors

*Jianliang Bai[1], Guojun Yuan[1], Xu Chen, Lu Zhang, Yaqing Zhu, Xinyu Wang, and Lili Ren**

Supporting Information

Simple Strategy for Scalable Preparation Carbon Dots: RTP, Time-dependence Fluorescence and NIR Behaviors

*Jianliang Bai, Guojun Yuan, Xu Chen, Lu Zhang, Yaqing Zhu, Xinyu Wang, Lili Ren**

Dr. J. Bai, Dr. G. Yuan, X. Chen, L. Zhang, Y. Zhu, Dr. X. Wang, Prof. L. Ren
School of Chemistry & Chemical Engineering
Southeast University
Nanjing 211189, China
E-mail: liliren@seu.edu.cn (L. Ren)

1. EXPERIMENTAL SECTION

Materials. *o*-phenylenediamine (OPD) and *p*-phenylenediamine (PPD) were purchased from Acros Organics. $AlCl_3 \cdot 6H_2O$ and $CuCl_2 \cdot 2H_2O$ were purchased from Aladdin. Ethanol (AR), methanol (AR) and dimethyl sulfoxide (AR) were purchased from Sinopharm Chemical Reagent Co.,Ltd (Shanghai, China). Deionized water (18.1 MΩ cm) was obtained using the Milli-Q water purification equipment.

Characterization. Transmission electron microscopy (TEM) images were obtained using a Talos F200X microscope operating at 200 kV. Fourier transform infrared (FT-IR) spectra were recorded using a Bruker Tensor 27 ATR-FTIR spectrometer with KBr pellets. UV-vis absorption spectra were recorded using a Shimadzu UV-2600 spectrometer. Photoluminescence (PL) spectra were obtained using a Horiba Fluoromax-4 fluorescence spectrophotometer. X-ray photoelectron spectroscopy (XPS) was conducted using a Thermo ESCALAB 250XI instrument. The FL QY and RTP decay curves of M-CDs were measured using a Fluorolog-3 spectrofluorometer. The RTP QY of M-CDs powder were measured using a C11347-11 spectrofluorometer. ^{1}H NMR spectrum was recorded on a BRUKER AMX 600 MHz instrument.

Preparation of M-CDs. In a typical synthesis of M-CDs, 0.54 g OPD and 0.08 g $AlCl_3 \cdot 6H_2O$ were ground by pestle milling for ten minutes in agate mortar. Then the mixed powder was transferred moved to an autoclave (20 mL) for heating 12 h at 200 °C. After cooled to room temperature, the obtained carbonized powders possess blue emission under a UV lamp (365 nm), yellow room-temperature phosphorescence after

ceasing the UV irradiation. And then powders were washed with deionized water three times to remove impurities, and the remnants were dried in an oven with 60 °C. In the end, the dried powders were dissolved into DMSO to achieve time-dependence fluorescence CDs for further characterizations. The reaction volume can be flexibly scaled up or down, thus the CDs should hold great potential for large-scale synthesis. In order to verify the reliability of the Water-Washing method, M-CDs are purified by column chromatography for comparison, and the M-CDs obtained by this method are named Column Chromatography M-CDs. The detailed operation process is as follows: The crude products of M-CDs were purified with a silica column chromatography using methanol as eluent. After removing solvent, the resultant M-CDs further dried at 40 °C for 24 h under vacuum. As shown in **Figure S17**, UV-Vis and FL spectra of Water-Washing M-CDs and Column Chromatography M-CDs in DMSO solutions show that their compositions are consistent. Hence, the Water-Washing method is considered as reliable as column chromatography. The yield of M-CDs obtained by Water-Washing method is about 71%, while that obtained by Column Chromatography method is about 59%.

Synthesis route of CDs 1-3. First, 0.54 g of OPD was dissolved in 35 mL of ethanol; then, the solution was transferred into 50 mL Teflon-lined stainless-steel autoclave. After being heated at 200 °C for 12 h and then cooled to room temperature, the obtained solution was purified *via* silica column chromatography using ethanol as the eluent. Afterward, CDs samples with different fluorescence colors (CDs-1: red; CDs-2: yellow; CDs-3: green) will be obtained.

Synthesis route of CDs-4. 0.020 g PPD was dissolved in 10 mL of deionized water and stirred for about 10 min. The mixed solution was transferred to a 20 mL Teflon-lined stainless-steel autoclave and heated at 200 ℃ for 2 h. After the reaction was complete, a cloudy solution with a small amount of precipitate was obtained, which was purified by silica column chromatography using ethanol as the eluent.

Synthesis route of CDs 5-6. First, 0.54 g PPD and 0.08 g $AlCl_3 \cdot 6H_2O$ were dissolved in 35 mL of ethanol; then, the solution was transferred into 50 mL Teflon-lined stainless-steel autoclave. After being heated at 200 °C for 12 h and then cooled to room temperature, the obtained solution was purified *via* silica column chromatography using ethanol as the eluent. Afterward, CDs samples with different fluorescence colors (CDs-5: red; CDs-6: green) will be obtained.

Synthesis route of M'-CDs. 0.54 g OPD and 0.056 g $CuCl_2 \cdot 2H_2O$ were ground by pestle milling for ten minutes in agate mortar. Then the mixture was transferred to a 20 mL autoclave for heating 12h at 200℃. After that, the autoclave was taken out and cooled to room temperature naturally. The obtained carbonized powders possess weak blue emission under a UV lamp (365 nm), weak yellow room-temperature phosphorescence after ceasing the UV irradiation. And then powders were washed with deionized water three times to remove impurities, and the remnants were dried in an oven with 60 ℃.

2. The evidence of hydrogen bonds among carbon nanoparticles.

In the solvent-free process of preparing M-CDs, we fully consider how to create hydrogen bonds and other non-covalent interactions among CDs, so as to ensure the realization of solid-state fluorescence and room temperature phosphorescence (RTP) of M-CDs. If the M-CDs are purified by dialysis or column chromatography, the hydrogen bonds among CDs will be destroyed directly. As shown in **Figure S6a**, the obtained M-CDs powder (by using Column Chromatography method) is no longer emitted blue fluorescence under a UV lamp (365 nm). We compared the FT-IR spectra of M-CDs purified by using Water-Washing and Column Chromatography methods. As shown in **Figure S6b**, the FT-IR spectra of M-CDs purified by Water-Washing (one time), Water-Washing (three times) and Column Chromatography methods have similar shape. However, the FT-IR spectra of M-CDs (Water-Washing three times and Column Chromatography) have obvious characteristic peaks at 3432 cm^{-1} attributed to the stretching vibrations N-H and O-H.[1] The FT-IR spectrum of M-CDs (Water-Washing one time) has obvious characteristic peaks at 3392 cm^{-1} attributed to the stretching vibrations N-H and O-H. The reason is that the formation of hydrogen bond makes the electron cloud density average, which reduces the stretching vibration frequency and causes the peak of proton donor to shift to low wavenumber.[2-5] This result is consistent with the hypothesis we proposed in the manuscript. At the same time, it provides reliable evidence for the dispersion induced red-shift phenomenon discussed in the following.

REFERENCES

(1) Gong, X.; Hu, Q.; Paau, M. C.; Zhang, Y.; Shuang, S.; Dong, C.; Choi, M. M. F.; Red-green-blue fluorescent hollow carbon nanoparticles isolated from chromatographic fractions for cellular imaging. *Nanoscale* **2014**, *6*, 8162-8170.

(2) Rozenberg, M.; Loewenschuss, A.; Marcus, Y. An empirical correlation between stretching vibration redshift and hydrogen bond length. *Phys. Chem. Chem. Phys.* **2000**, *12*, 2699-2702.

(3) Steiner, T. The Hydrogen Bond in the Solid State. *Angew. Chem. Int. Ed.* **2002**, *41*, 48-76.

(4) Li, Q.; Zhou, M.; Yang, M.; Yang, Q.; Zhang, J.; Shi, J. Induction of long-lived room temperature phosphorescence of carbon dots by water in hydrogen-bonded matrices. *Nat. Commun.* **2018**, *9*, 734.

(5) Gao, Y.; Zhang, H.; Jiao, Y.; Lu, W.; Liu, Y.; Han, H.; Gong, X.; Shuang, S.; Dong, C. Strategy for Activating Room-Temperature Phosphorescence of Carbon Dots in Aqueous Environments. *Chem. Mater.* **2019**, *31*, 7979-7986.

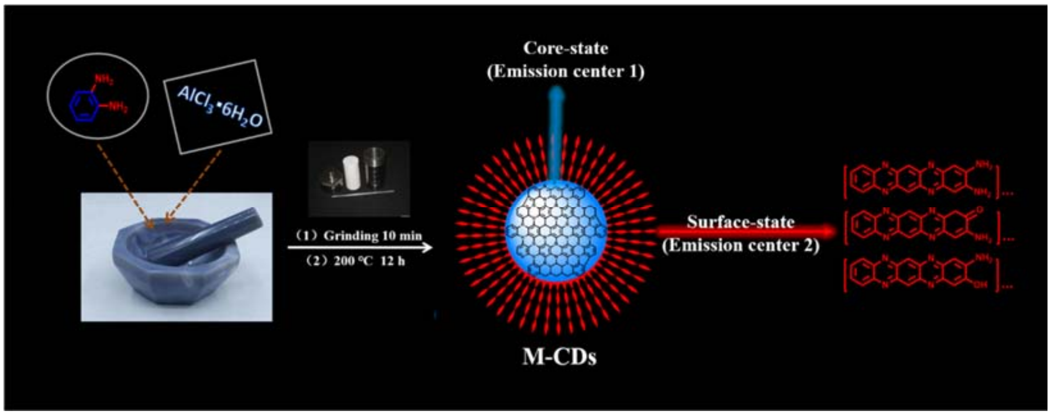

Figure S1. Schematic representation of synthesis process of M-CDs through the solvent-free $AlCl_3$ catalytic assistant strategy.

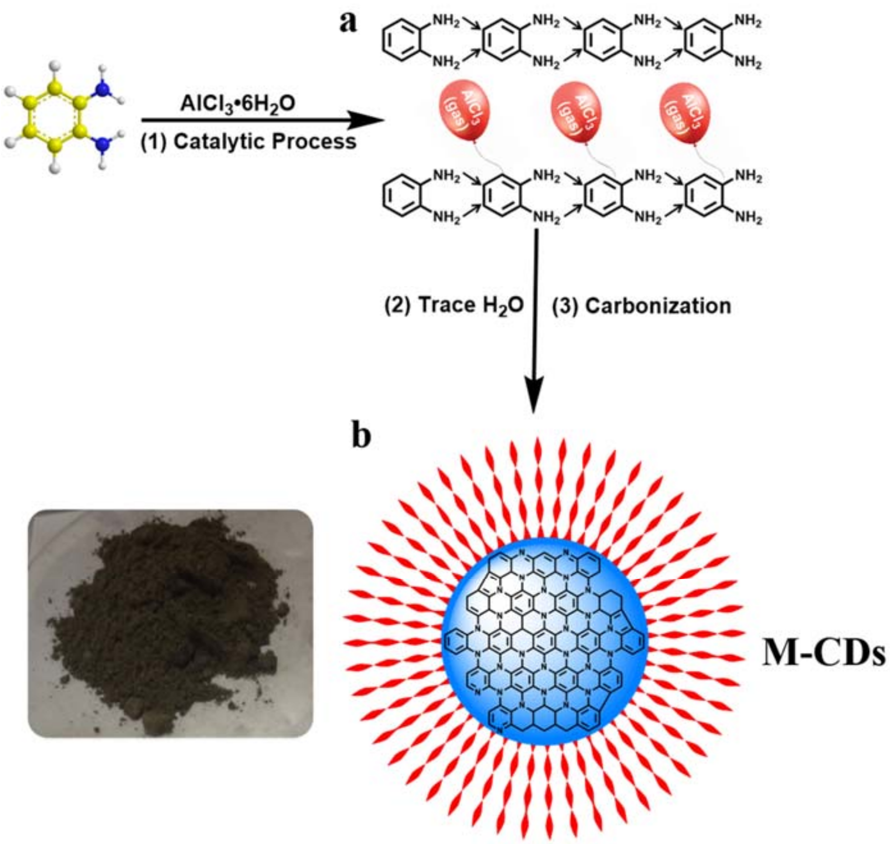

Figure S2. The detailed formation process of the M-CDs.

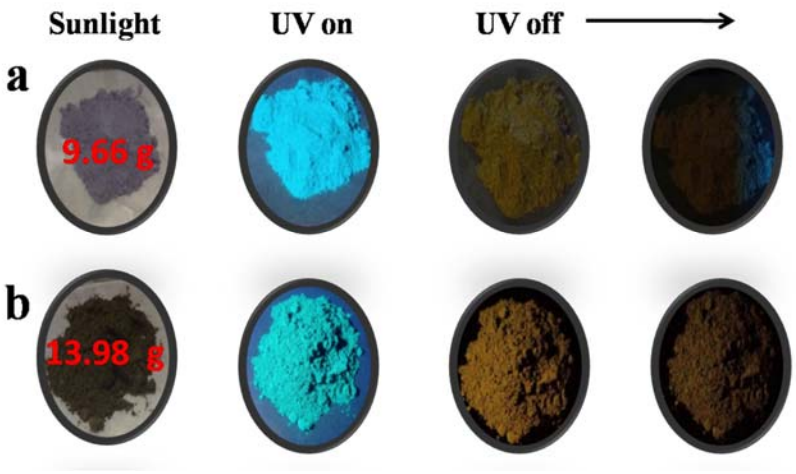

Figure S3. Schematic illustration of the mass production of M-CDs by solvent-free catalytic assistant strategy (a) 9.66 g; (b) 13.98 g.

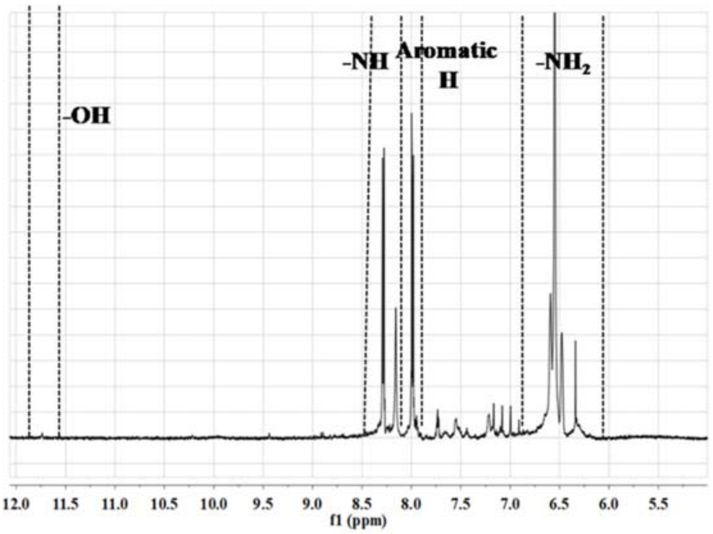

Figure S4. ^{1}H NMR spectrum of the M-CDs.

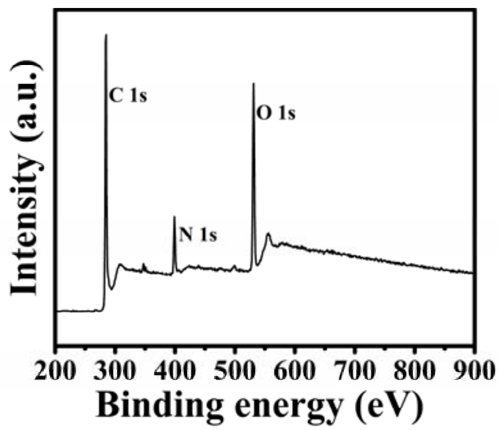

Figure S5. XPS spectrum of the M-CDs.

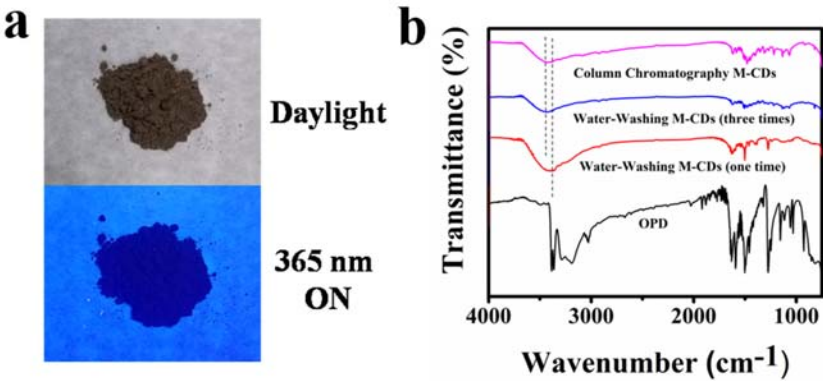

Figure S6. (a) Images of the M-CDs (by using Column Chromatography method) powder under daylight, UV lamp (365 nm) ON. (b) Comparison of FT-IR of Column Chromatography M-CDs, Water-Washing (three times) M-CDs, Water-Washing (one time) and OPD.

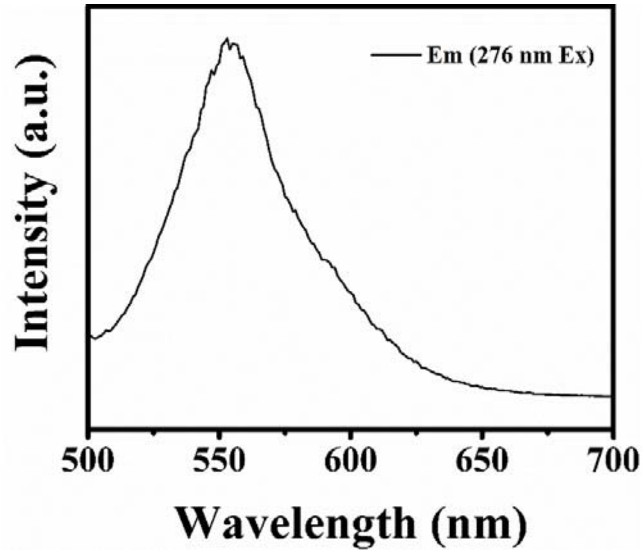

Figure S7. The phosphorescence emission spectrum of the M-CDs powder under excitation at 276 nm.

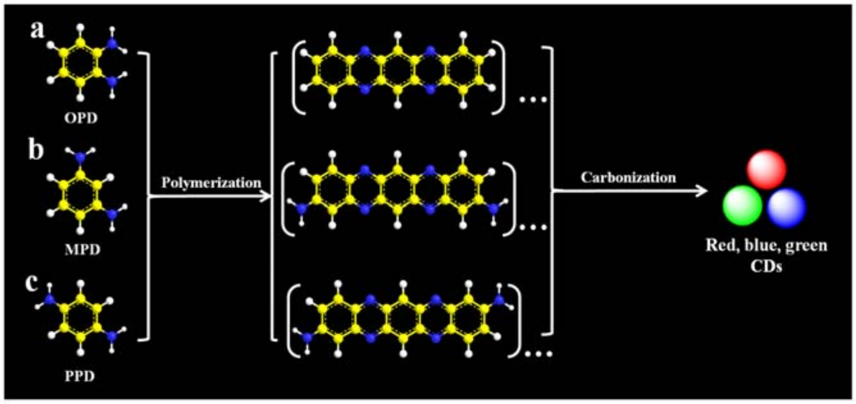

Figure S8. Preparation of the red, green, blue PL CDs from three different phenylenediamine isomers.

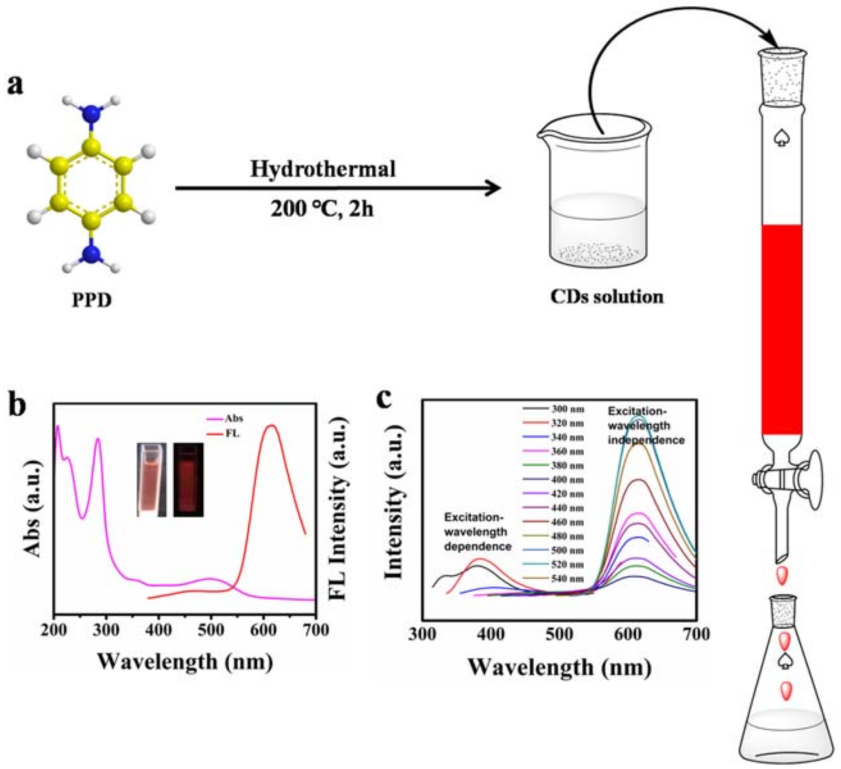

Figure S9. (a) One-pot synthesis and purification route for CDs-4. (b) UV-Vis absorption and FL spectra for CDs-4. (c) The FL emission spectra of the CDs-4 dispersed in CH_3CH_2OH at different excitation wavelengths (i.e., 300, 320, 340, 360, 380, 400, 420, 440, 460, 480, 500, 520 and 540 nm) under ambient conditions.

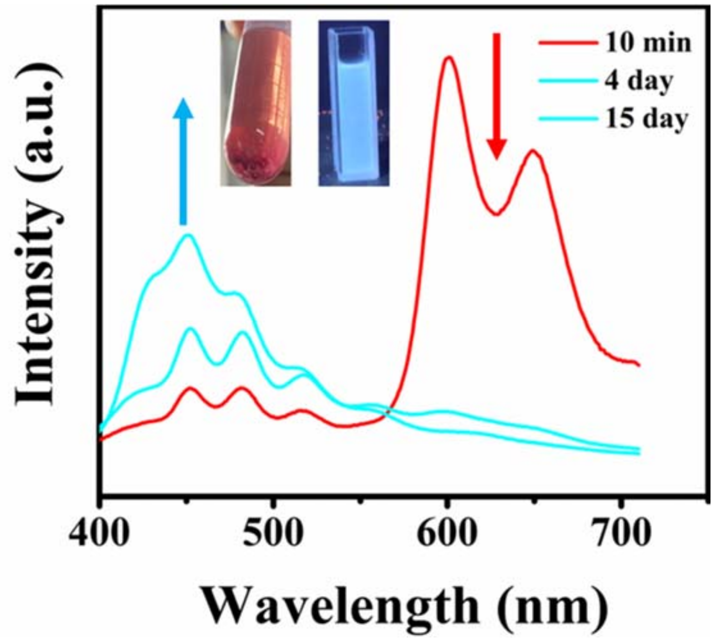

Figure S10. FL emission spectra of CDs-1 (dispersed in ethanol solution) recorded immediately and after 4 days, 15 days (Inset: Illuminating photographs under daylight and 365 nm UV irradiation).

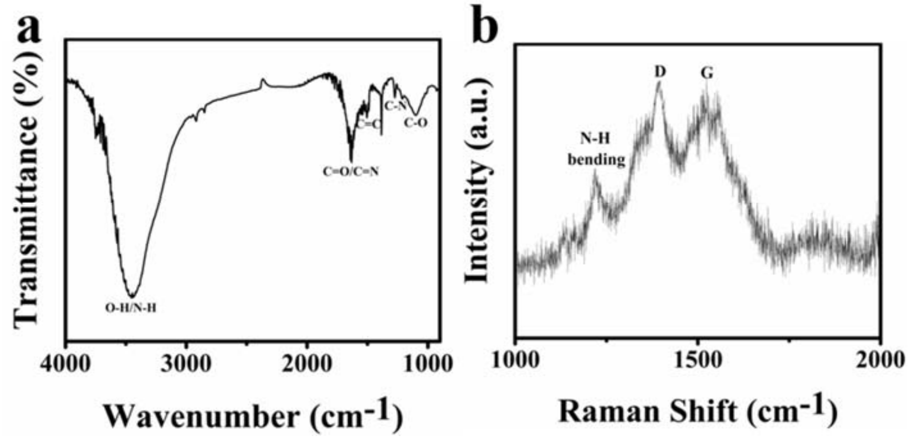

Figure S11. (a) FT-IR spectrum of CDs-1. (b) Raman spectrum of CDs-1.

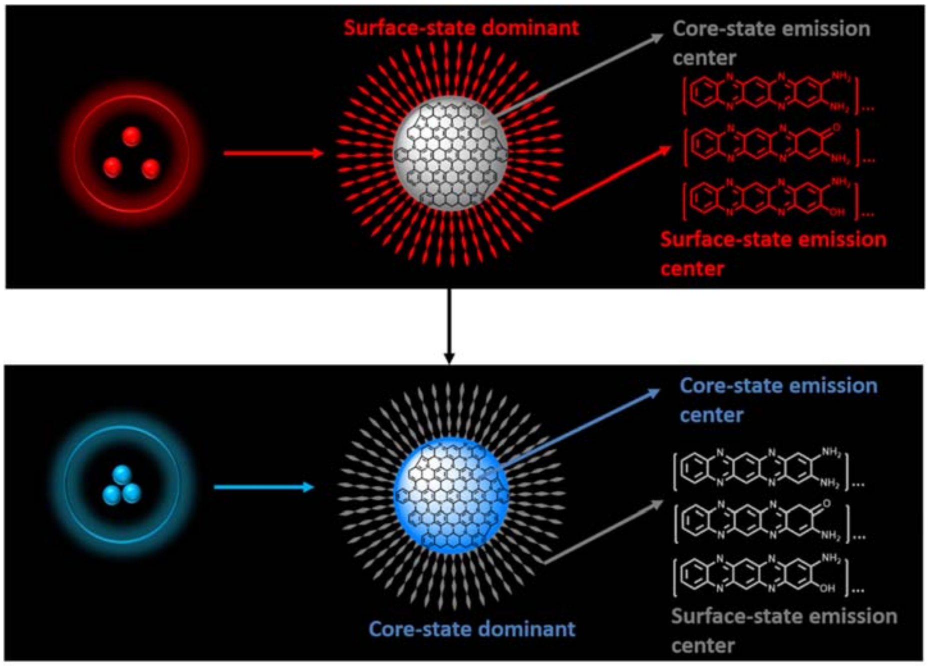

Figure S12. Schematic illustration of the process of fluorescence change of the CDs-1 from dispersed state to aggregated state.

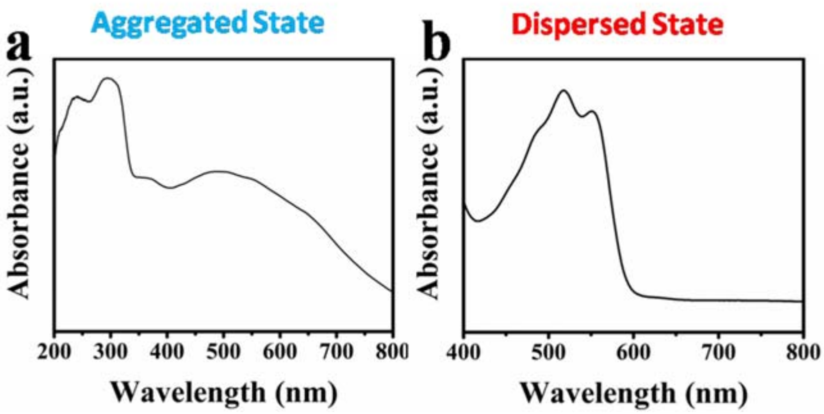

Figure S13. (a) Absorption spectrum of M-CDs (aggregated state). (b) Absorption spectrum of M-CDs (dispersed state).

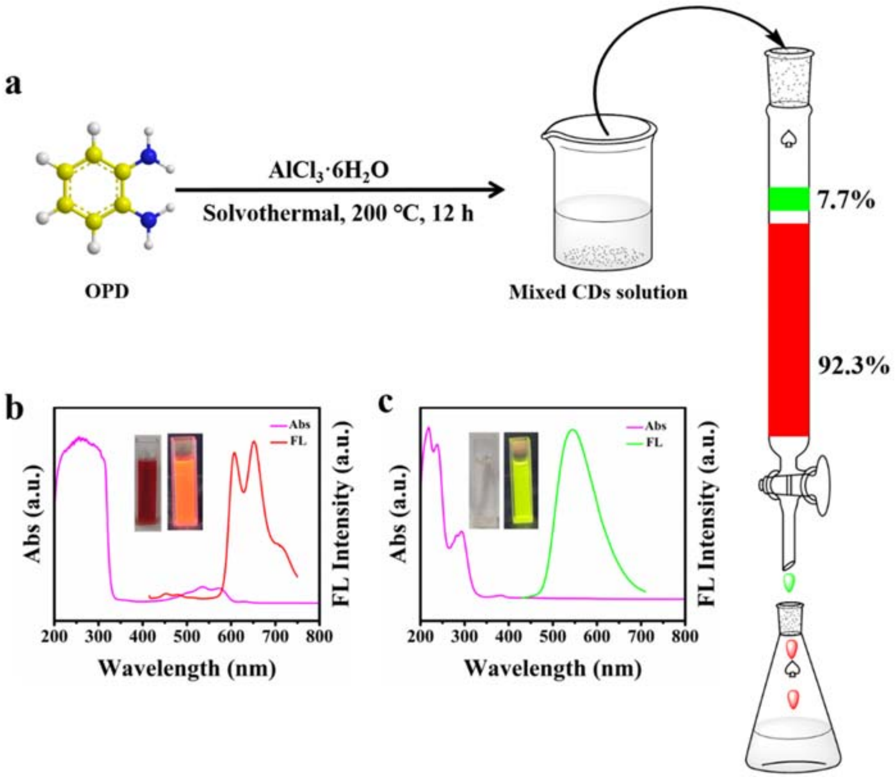

Figure S14. One-pot synthesis and purification route for CDs-5-6 with distinct FL characteristics. (b-c) UV-Vis absorption and FL spectra for CDs-5-6.

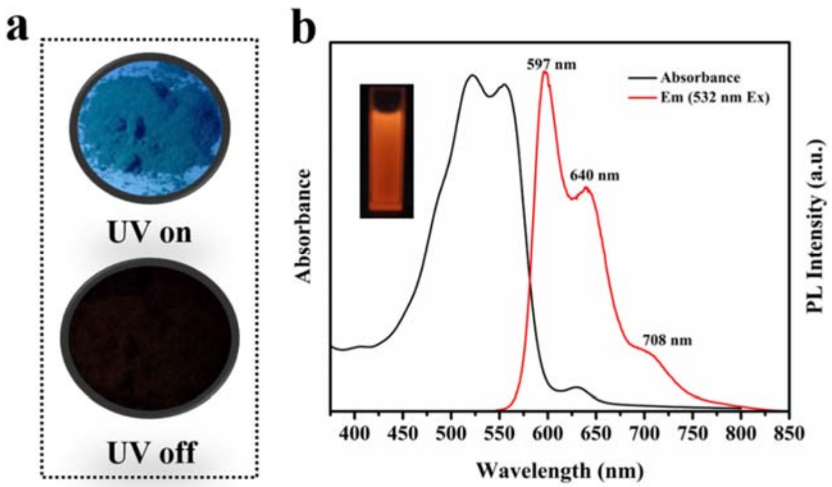

Figure S15. (a) Photographs of the M’-CDs powder under 365 nm UV lamp on and off, respectively. (b) UV-Vis absorption and FL spectrum for M’-CDs.

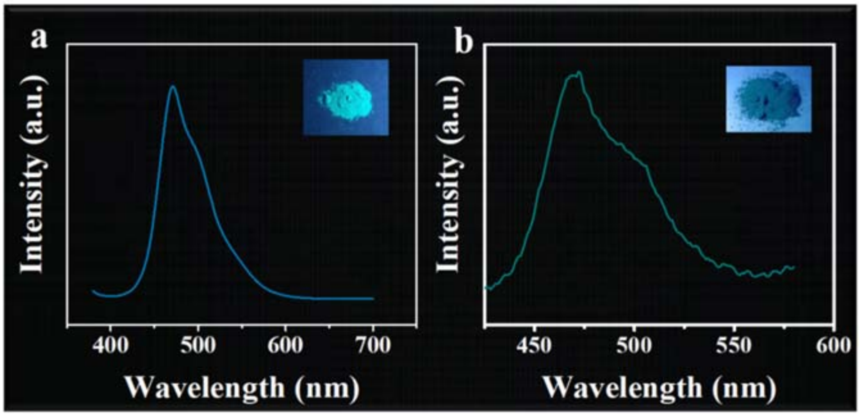

Figure S16. (a) The FL emission (λ_{ex} = 365 nm) spectrum of M-CDs powder. (b) The FL emission (λ_{ex} = 365 nm) spectrum of M'-CDs powder.

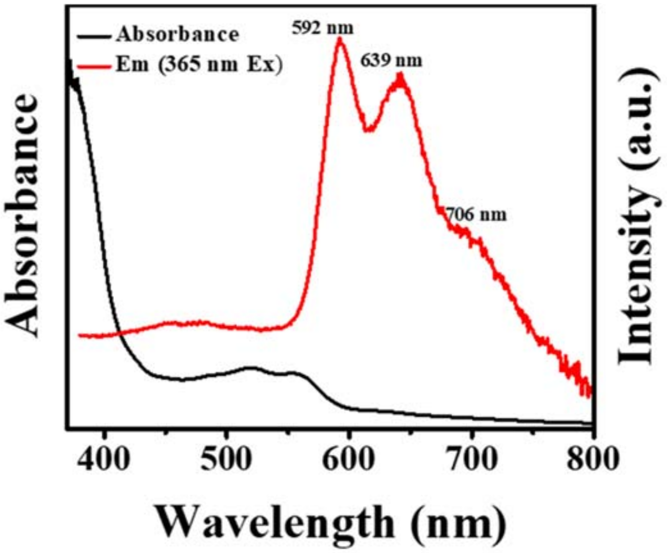

Figure S17. UV-Vis absorption and FL spectra for M-CDs (Column Chromatography method).

Table S1. Fitting parameters of the afterglow decay curves of M-CDs under excitation at 276 nm.

CDs	$\tau 1$ (ms)	A1(%)	$\tau 2$ (ms)	A2 (%)	$\tau 3$ (ms)	A3 (%)	τ_{avg}(ms)	χ^2
M-CDs	5.2009	0.89	95.7788	13.18	251.8561	85.93	243.2	1.250

Table S2. The QYs of FL and RTP for the M-CDs.

	Sample	Excitation wavelength	QYs (%)
FL	M-CDs Powder	365 nm	3.2
RTP	M-CDs Powder	365 nm	2.8
FL	M-CDs in DMSO	490 nm	57
